# Supplementary material for: MicroRNA-Mediated Downregulation of HMGB2 Contributes to Cellular Senescence in Microvascular Endothelial Cells
Source: Cells. 2022 Feb 8;11(3):584. doi: 10.3390/cells11030584 (PMC8834370; doi:10.3390/cells11030584)
Supplement: Supplementary file 1 [file cells-11-00584-s001.zip › Supplementary/Supplementary tableS1-S3.pdf]

Table S1. Primer sequences used for qRT-PCR.

Gene	Primer sequences
PPIA/CYPA	5′-CCCACCGTGTTCTTCGACAT-3′
	5′-CCAGTGCTCAGAGCACGAAA-3′
RPL13A	5′-CCTGGAGGAGAAGAGGAAAGAGA-3′
	5′-TTGAGGACCTCTGTGTATTTGTCAA-3′
HMGB2	5′-CTTGGCACGATATGCAGCAA-3′
	5′-CAGCCAAAGATAAACAACCATATGA-3′
CDKN1A/p16	5′-CATAGATGCCGCGGAAGGT-3′
	5′-CCCGAGGTTTCTCAGAGCCT-3′
CDKN1A/p21	5′-CTGCGCCAGCTGAGGTGTGAG-3′
	5′-GCCGCATGGGTTCTGACGGA-3′
miR-23a-3p	5′-ATCACATTGCCAGGGATTTCC-3′
miR-23b-3p	5′-ATCACATTGCCAGGGATTACC-3′
miR-181a-5p	5′-AACATTCAACGCTGTCGGTGAGT-3′
miR-181b-5p	5′-AACATTCATTGCTTGTCGGTGGGT-3′
miR-221-3p	5′-AGCTACATTGTCTGCTGGGTTTC-3′
miR-222-3p	5′-AGCTACATCTGGCTACTGGGT-3′
mRQ 3′	Reverse primers of miRNAs were provided in Mir-X™ miRNA First-Strand Synthesis Kit

Table S2. The sequences of siRNA and miRNA mimics.

Gene	Primer sequences
siControl	5′-CCUACGCCACCAAUUUCGUUU-3′
siHMGB2 #1	5′-CUGAACAUCGCCCAAAGAU-3′
siHMGB2 #2	5′-AGUGAACACCCUGGCCUAU-3′
miRControl	5′-CCUCGUGCCGUUCCAUCAGGUAGUU-3′
hsa-miR-23a-3p	5′-AUCACAUUGCCAGGGAUUUCC-3′
hsa-miR-23b-3p	5′-AUCACAUUGCCAGGGAUUACC-3
hsa-miR-181a-5p	5′- AACAUUCAACGCUGUCGGUGAGU-3′
hsa-miR-181b-5p	5′-AACAUUCAUUGCUGUCGGUGGGU-3′
hsa-miR-221-3p	5′-AGCUACAUUGUCUGCUGGGUUUC-3
hsa-miR-222-3p	5′-AGCUACAUCUGGCUACUGGGU-3′

Table S3. The miRNA target prediction platform used in this study.

Platform	URL
miRSystem	http://mirsystem.cgm.ntu.edu.tw/
miRDB	http://www.mirdb.org/index.html
microT-CDS	http://diana.imis.athena-innovation.gr/DianaTools/index.php?r=microT_CDS/index
TarBase_v.8	https://carolina.imis.athena-innovation.gr/diana_tools/web/index.php?r=tarbasev8%2Findex
miRWalk	http://mirwalk.umm.uni-heidelberg.de/
